# Tension gastrothorax: acute life-threatening manifestation of late onset congenital diaphragmatic hernia (CDH) in children

**DOI:** 10.1186/s13049-015-0129-8

**Published:** 2015-06-24

**Authors:** Pål Aksel Næss, Joachim Wiborg, Kristin Kjellevold, Christine Gaarder

**Affiliations:** Department of Traumatology, Oslo University Hospital Ulleval, Nydalen postbox 4956, N-0424 Oslo Norway; Department of Gastrointestinal and Pediatric Surgery, Oslo University Hospital Ulleval, Nydalen, Norway

**Keywords:** Tension gastrothorax, Laparotomy, Children

## Abstract

Tension gastrothorax in children is a life-threatening condition and presents dramatically with acute and severe respiratory distress. It develops when an intra-thoracic stomach herniated through a diaphragmatic defect is massively distended by trapped air and/or fluid causing mediastinal displacement. Tension gastrothorax is often misinterpreted as tension pneumothorax and managed as such leading to increased morbidity and mortality. We present a child with tension gastrothorax and a literature review of this phenomenon.

Immediate clinical and radiographic evaluation should lead to accurate diagnosis followed by emergency decompression of the stomach before laparotomy with reduction of herniated viscera and repair of the diaphragmatic defect.

## Background

Tension gastrothorax presents dramatically with acute and severe respiratory distress. It develops when the stomach herniated through a left-sided diaphragmatic defect into the thorax is massively distended by trapped air and/or fluid. The term tension gastrothorax first appeared in the literature in 1984 as a complication of traumatic rupture of the diaphragm in an adult [1]. However, in childhood this phenomenon is dominantly caused by a herniation of the stomach through a posterolateral congenital diaphragmatic defect [2–4]. This article focuses on symptoms, diagnosis and treatment of this life-threatening condition in children based on a case report and review of the literature.

## Case presentation

### Case report

**Fig. 1:**
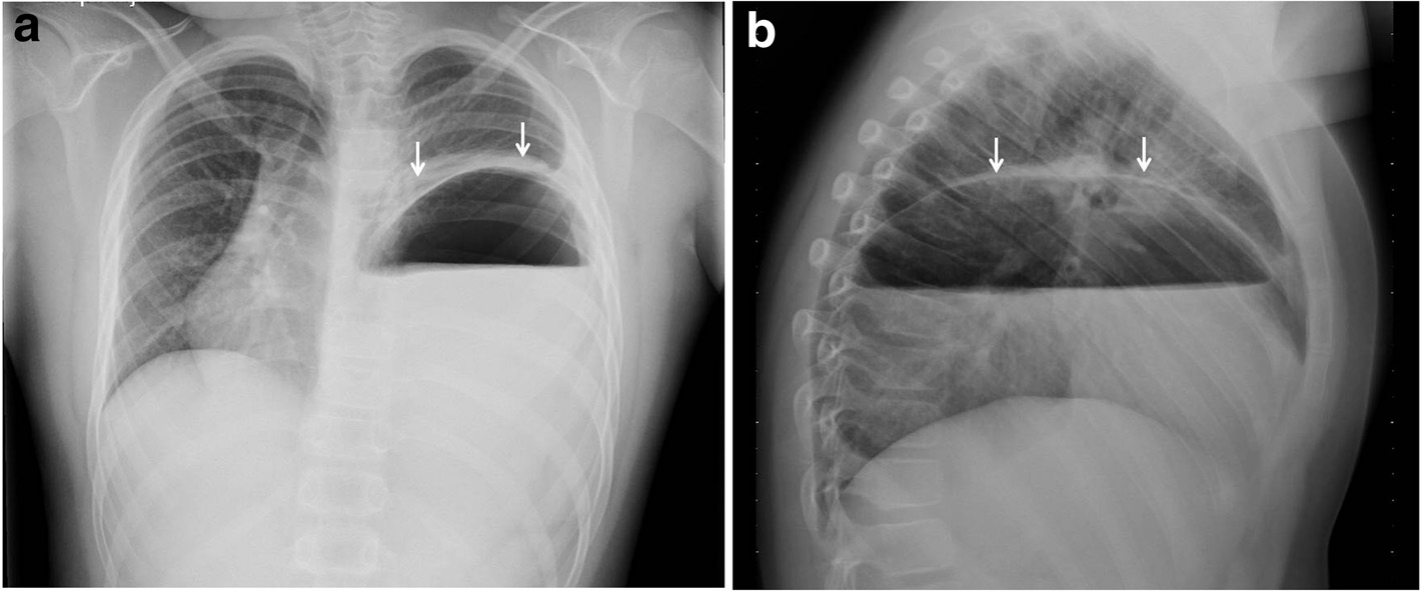
Anteroposterior (**a**) and lateral (**b**) chest radiographs with large air-fluid level in left hemithorax. Note superior rim formed by stomach wall and compressed lung (arrows) and mediastinal shift to the right

**Fig. 2:**
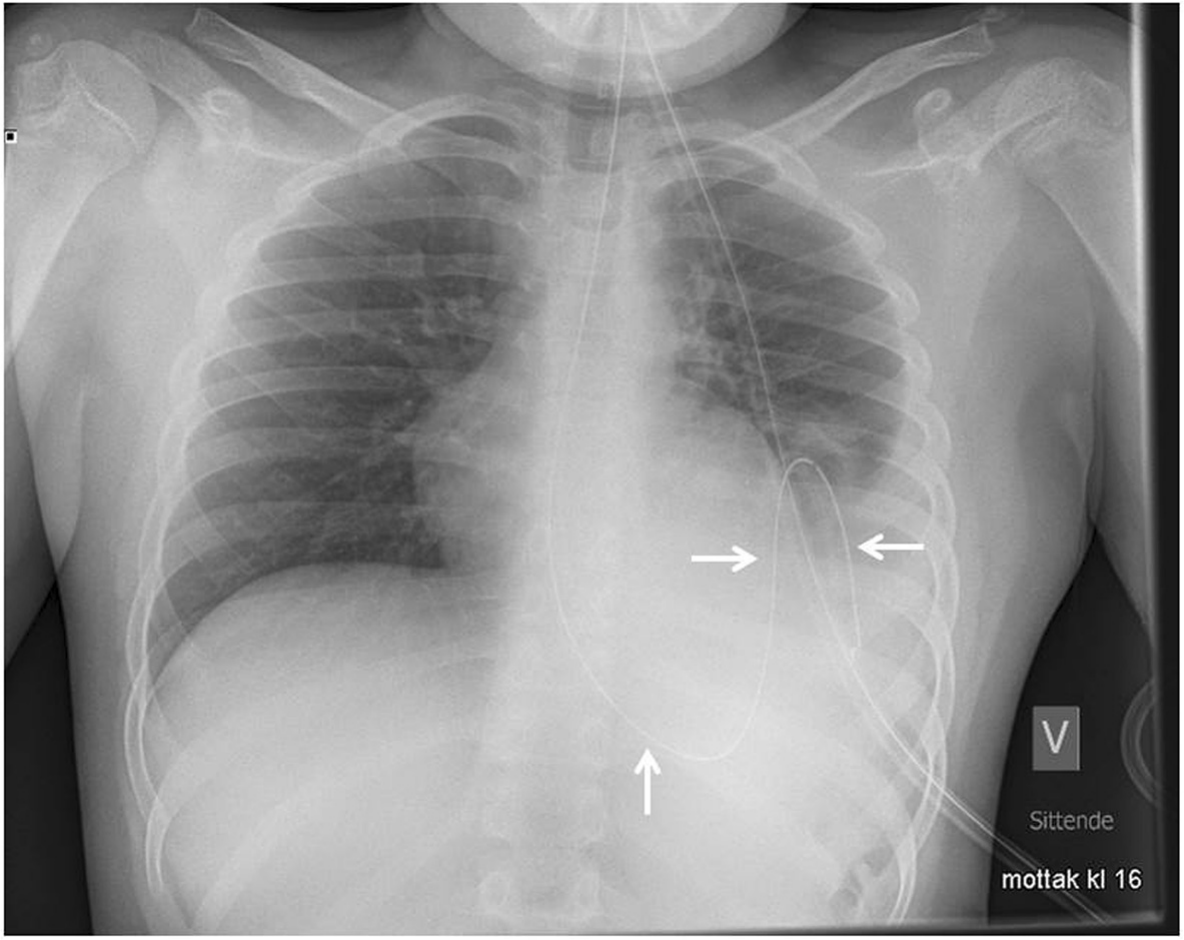
Chest x-ray after gastric decompression. Note the normalization of the mediastinal shift and the gastric tube as it curves back into the left hemithorax (arrows) confirming the intrathoracic position of the stomach

**Fig. 3:**
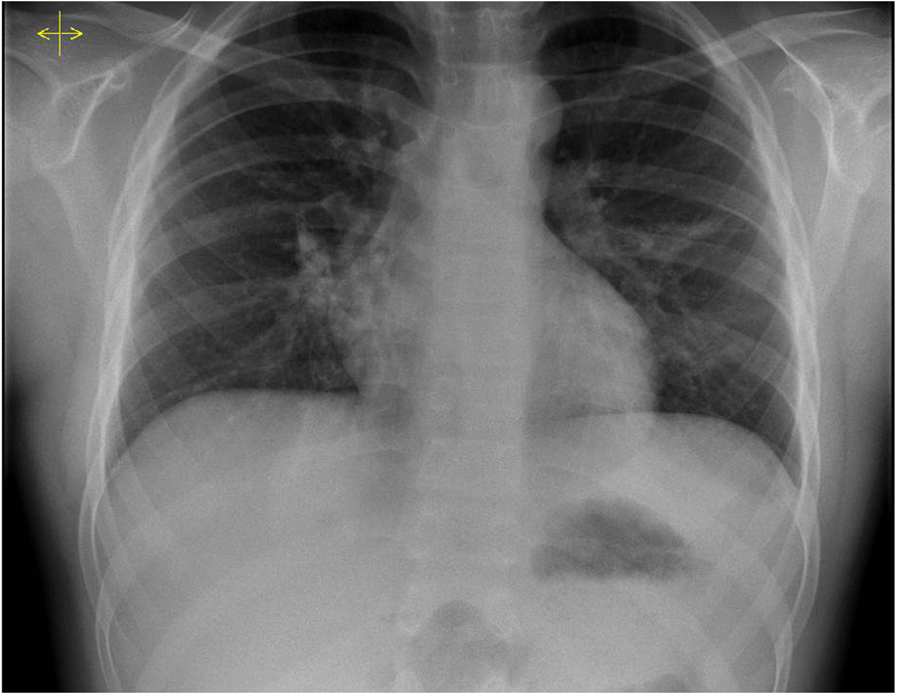
Chest x-ray at follow-up 2 months after surgical repair of the diaphragmatic defect. Note normal position of the stomach bubble and the normal diaphragmatic contour

A previously healthy 9-year-old boy presented to the emergency department with a 6 h history of left chest pain and increasing respiratory distress. His respiratory rate was 40/min and heart rate was 105/min, cardiac auscultation was unremarkable, auscultation of lung fields revealed diminished breath sound over the left side. A chest x-ray (Fig. 1) showing a large air-fluid level in the left hemithorax with shift of the mediastinum to the right was interpreted as tension gastrothorax. Prompt insertion of a nasogastric tube with evacuation of air and 800 ml of gastric content led to immediate relief of symptoms. Subsequent chest x-ray showed normalization of the mediastinal shift and the nasogastric tube was found to curve back into the left hemithorax (Fig. 2). On the same day, the patient underwent repair of a 5 × 5 cm posterolateral defect in the left diaphragm after the stomach and spleen were repositioned in the abdominal cavity. The postoperative course was uneventful and he was discharged home 7 days after surgery. He remained well at follow-up 2 and 8 months later and chest x-ray was normal (Fig. 3).

## Discussion

Tension gastrothorax describes mediastinal shift by a distended intrathoracic stomach herniated through a congenital or acquired diaphragmatic defect [1–3]. Most pediatric cases are left-sided and the stomach has herniated through a congenital posterolateral (Bochdalek) defect [3].

Congenital diaphragmatic hernia (CDH) occurs in 1 in 2500–4000 live births and the vast majority are diagnosed prenatally or shortly after birth due to respiratory distress [5, 6]. However, approximately 10 % of patients with CDH present later in life [2, 7, 8]. Late presentation of CDH presents diagnostic difficulty due to its rarity and misleading symptoms and signs [2, 3, 7, 8]. In childhood the clinical presentation varies from nonspecific symptoms like chest pain and abdominal pain, failure to thrive and recurrent pulmonary infection to severe respiratory distress and eventually circulatory collapse caused by a fully developed tension gastrothorax [2, 3, 7–9].

A likely chain of pathophysiology events leading to tension gastrothorax is described by Horst et al. [2]. At some point increased abdominal pressure herniates the stomach through a preexisting defect in the diaphragm. Then tension gastrothorax may occur at any time when the stomach suddenly fills with air, fluid or food through a one-way valve mechanism created by abnormal angulation of the gastroesophageal junction combined with gastric outlet obstruction caused at the level of the diaphragm [2, 3].

The clinical picture of tension gastrothorax with acute respiratory distress and reduced or absent breath sounds in the left hemithorax in an otherwise well child has commonly been mistaken for a tension pneumothorax and managed as such leading to increased morbidity and mortality [3, 8–10].

The proper interpretation of the chest x-ray becomes crucial when differentiating between the above mentioned diagnoses. The radiological findings of a tension gastrothorax include: a large air-filled structure with or without a fluid level in the left hemithorax, a superior rim formed by compressed ipsilateral lung and stomach wall, lack of a stomach bubble in the left upper quadrant, the left hemidiaphragm will be poorly defined as well as mediastinal shift to the right (Fig. 1) [2, 3, 10, 11].

Although mediastinal shift to the right is evident in a left-sided tension pneumothorax, the following features will allow clear-cut distinction from tension gastrothorax: the entire left lung is centrally compressed and all surrounded by intrapleural air, the lateral sinus is free and the left (depressed) hemidiaphragm well-defined [2, 3, 11]. Moreover, a tension pneumothorax in an otherwise healthy child is an uncommon event [10].

The management of tension gastrothorax is immediate placement of a large-bore naso- or orogastric tube to decompress the dilated stomach [2, 3, 8, 12]. Intubation of the intrathoracic segment may be difficult [10]. The position of the tube on a chest x-ray as it curves back into the chest is a very helpful and virtually diagnostic finding (Fig. 2) [11]. Instant clinical improvement should occur after stomach decompression [10]. If this maneuver fails, transthoracic needle decompression of the stomach in a lower intercostal space guided by the chest x-ray is recommended [2, 3]. If deflation of the stomach is not accomplished the mediastinal shift can impair venous return and lead to cardiac arrest [3, 12–14].

Definitive management after initial resuscitation in this emergency is operative repair. Laparotomy is the access of choice [3, 4]. It allows quicker reduction and inspection of the abdominal viscera and easy repair of the diaphragmatic defect [2, 14]. In an otherwise healthy child, as in the presented case, an uneventful recovery can be expected [2].

## Conclusions

Although rare, tension gastrothorax must be included in the differential diagnosis in a previously healthy child with acute onset of severe respiratory distress to allow prompt life-saving action. Immediate clinical and radiographic evaluation leads to accurate diagnosis and should be followed by emergency decompression of the stomach before laparotomy with reduction of herniated viscera and repair of the diaphragmatic defect.

## Consent

Written informed consent was obtained from the legal guardians of the patient for publication of this Case report and the accompanying images. A copy of the written consent is available for review by the Editor-in-Chief of this journal.
